# Expectations, Tensions, and Brokerage: A Discourse Analysis of Community Engagement with Health Research in South Africa

**DOI:** 10.1002/eahr.60012

**Published:** 2025-03-14

**Authors:** Sonja Klingberg, Catherine E. Draper

**Affiliations:** ^1^ Postdoctoral researcher at SAMRC (South African Medical Research Council)/Wits Developmental Pathways for Health Research Unit in the Department of Paediatrics in the Faculty of Health Sciences at the School of Clinical Medicine at the University of the Witwatersrand; ^2^ Associate professor at SAMRC/Wits Developmental Pathways for Health Research Unit in the Department of Paediatrics in the Faculty of Health Sciences at the School of Clinical Medicine at the University of the Witwatersrand

**Keywords:** community engagement, research ethics, health research, community‐based health research, inequality, discourse analysis, South Africa

## Abstract

Research is increasingly claimed to be done in collaboration with communities, but community members may have entirely different expectations of engagement and research participation than what typically follows the logic of academia. In South Africa, intersecting inequalities further complicate relationships with communities and stakeholders. To understand how different actors view and construct the relationships between academic institutions and communities, we undertook a multiperspective discourse analysis. We conducted 11 in‐depth interviews with 12 participants categorized as researchers, community representatives, and community members. These interviews reflect three interconnected discourses: expectations, tensions, and brokerage. *Expectations* pattern intergroup dynamics, such as community members’ expectations of research benefits, while *tensions* primarily capture challenging relationships between different research actors. Our analysis also illustrates how, in the absence of comprehensive institutional support, community engagement relies on *brokerage* by community representatives, and how this reliance disproportionately burdens them. There is a need to lessen this ethical burden and invite community input without also burdening community members and representatives with the challenges of academia. Our findings also have wider relevance for community‐based health research because engagement practices are often hindered by institutional and structural factors.

Community engagement is a recommended practice in health research, building on democratic and ethical principles of involving intended beneficiaries in the conduct of research.^1^ There are many ways to approach community engagement, which can broadly be seen to encompass relationship‐building between research initiatives, communities, and stakeholders of some relevance to the research.^2^ While commitment to community engagement and its prioritization through funding have strengthened over the past few decades,^3^ there have also been critical discussions about the often tokenistic nature of community engagement in connection with health research. A recent realist review points to factors such as lack of institutional flexibility or support and training for researchers as some reasons current community engagement and participatory research practices may hinder the intended benefits of research or interventions.^4^

Challenges of community engagement are particularly important to consider in settings where the intersecting inequalities associated with academic research conducted with (or, perhaps more typically, in) disadvantaged communities heavily influence the potential for transformative or meaningful engagement.^5^ In our health research in mostly urban areas of South Africa, we have grappled with the complex layers of history, power dynamics, institutional logics, and motivations to do “good” through academic research despite academic and systemic structural forces that can overshadow such attempts.

Health research in many settings in South Africa still often reflect historical dynamics of predominantly white and/or foreign, highly educated, relatively wealthy, researchers doing research on and in predominantly Black and low‐income communities. In addition to academia and communities, an important group to consider is also the interlocutors or brokers—the research and project staff, fieldworkers, and community engagement officers “in between,” (here called community representatives) whose roles involve bridging the two worlds of academic research and community realities.^6^ Kalinga^7^ has written about this phenomenon in an illustratively named piece, “Caught Between a Rock and a Hard Place: Navigating Global Research Partnerships in the Global South as an Indigenous Researcher.” While Kalinga's focus is on the dynamics of international research collaborations that often make assumptions about the role of local Indigenous researchers as representatives of the communities involved in the research, we see very similar phenomena in the research carried out by local (but not local to the communities involved in research) South African research teams in contexts that vastly differ from the lived experiences of the researchers (differences due to historical and current intersecting inequalities of race, gender, education level, income, and more). In many research fields in South Africa, many senior researchers are still white and privileged from an economic and educational perspective, including in contrast to more junior research staff. These local and often white researchers can still feel like and/or be perceived as outsiders to the communities in which they work. This sense can be exacerbated by the involvement of international collaborators,^8^ particularly if they are also white and privileged.

To add further complexity in the South African context, there are frequently nongovernmental, not‐for‐profit, and/or community‐based organizations that play a critical role in health and human development, often standing in the gap where government services are failing. How researchers engage with such organizations can also reinforce entrenched power dynamics, and researchers may be perceived to be out of touch with the lived realities of communities, requiring the researchers to prove their credibility as equal partners. This topic of identity and positionality has been tackled by researchers in the early childhood development sector who grapple with how these power symmetries can be shifted through meaningful partnerships that acknowledge the intersectionality of privilege.^9^

As part of a wider research agenda around developing ethical and meaningful community engagement practices for our research in South Africa, we wanted to better understand how different stakeholders approach community engagement. As community engagement is

**We identified three main discourses that capture the dynamics and interconnectedness of the three participant groups’ perspectives on community engagement: expectations, tensions, and brokerage.**

inherently relational, it is important to consider its different meanings for the different parties involved. From our experiences, the diversity of research teams in South Africa also brings enormous opportunities and richness to the process, but problematic hierarchies are a reality that cannot easily be done away with despite best intentions and conscious efforts.

To move beyond our impressions and experiences of hierarchical dynamics, we set up a qualitative study employing discourse analysis to investigate the phenomenon in more detail and to develop recommendations or cautions for the wider research field that may be grappling with similar complexities in other global contexts. We chose a multiperspective approach and focused particularly on the social situatedness of discourses to unpack the complex power dynamics and hierarchies at play in the relationships built and maintained between academic institutions and historically oppressed and disadvantaged groups or communities in South Africa today. The aim of our study was thus to identify and analyze discourses about community engagement with health research in South Africa from the perspectives of different research stakeholders.

## Study Methods

We employed discourse analysis to generate an understanding of what community engagement means to different actors, and how they situate themselves in the dynamics of community‐research relationships. We approached discourse analysis from a constructivist standpoint, using critical and intersectional social theory about power and social structures to examine how participants representing different perspectives construct discourses about community engagement with research.^10^

Our study focused on health research in South Africa, particularly in the context of our own health research in predominantly low‐income urban settings. The study participants represented broad perspectives and multiple research settings and communities across this country. In South Africa, the linkage between academic researchers and communities can be managed through more formalized community advisory groups and engagement processes, partnerships with community‐based organizations, and study advisory groups made up of community members. Formalized community engagement structures tend to be more common in rural areas (often former “homelands” under apartheid) where engaging with traditional and/or tribal authorities is an essential part of the research process. Across settings, community leaders may be a mix of local government representatives, faith leaders, and other community activists. While funders of scientific research are increasingly requiring researchers to have established community engagement plans and support these within grant budgets, historically, these plans tend to be project‐based and hence difficult to sustain beyond the lifespan of a project.

We recruited South African research stakeholders from three categories: researchers, community representatives (who represent communities but work in research), and community members (also seen as activists or “actives,” i.e., somehow working for their communities). The decision to target community members who are activists or actives rather than community members in general was based on their involvement in promoting well‐being from a community‐centered perspective, specifically without an academic lens or logic.

While we categorized participants based on their representative perspectives, we did not take their representation to mean speaking for their entire group, but rather to situate their perspectives as being linked to the roles they held or played in relation to community engagement and research. Participants who were researchers were, or had been, working and actively involved in academic research that included community engagement in South Africa. Participants who were community representatives were employed in academic research institutions as community engagement officers or project staff and tended to be from the same communities where they were doing research. Participants representing community members were involved in health‐related community activism, community‐based organizations, and local government.

The sample size was not decided in advance but was determined by reaching a reasonable balance of participants representing different perspectives (research, community, and a combination of the two), and was based on considerations around data being saturated when sufficiently rich for theorizing and generating meaning.^11^ Sample characteristics are presented in table 1 in accordance with sociodemographic terminology used in South Africa and based on participants’ self‐identification in terms of population group and gender.

**Table 1:** Summary of Participant Characteristics

Participant characteristics* (n = 12)	Frequency
Represents research perspectives	3
Represents community perspectives	4
Represents both perspectives	5
Women	6
Men	6
Black African descent	8
South African Coloured descent	2
White South African descent	2

* Sociodemographic terminology used in South Africa.

SK interviewed 12 participants in English between March 2021 and August 2023 through a combination of in‐person and remote in‐depth interviews according to participants’ preferences and South African Covid‐19 restrictions in place at the time of each interview. Two of the participants were interviewed together based on their preference. We obtained ethical approval from the Human Research Ethics Committee (Medical) of the University of the Witwatersrand.

After receiving information about the study and having opportunities to ask questions, all participants gave written consent for being interviewed and for interviews to be sound recorded. In the case of in‐person interviews, transportation reimbursement and refreshments were provided, and all participants received a gift voucher valued at ZAR100 (approximately £5 or $5). Sound recorded interviews were transcribed verbatim and transcripts were anonymized.

Our discourse analysis draws on earlier work involving interview data from multiple stakeholder perspectives with a focus on ethical research and related roles and dilemmas.^12^ The process began with reading and immersion in a groupwise pattern,^13^ based on our strong impressions from the data collection phase showing that the three different groups of stakeholders brought distinct, complementary, insights to our understanding of community engagement in the context of health research in South Africa. Next, SK coded each transcript for manifest content in an inductive, data driven way, using MAXQDA qualitative analysis software (release 20.4.2) to aid the process. SK then organized these codes so that patterns across transcripts within each group were captured, still keeping the code systems separate between the three groups. By this point, the similarities and connections between the groups were familiar to us, and we were able to generate initial discourses that captured overarching patterns while still bearing in mind that the discourses manifested differently in each of the groups. We had extensive discussions about the discourses and the nuances and characteristics we considered important from each group. As a result, we further refined the discourses, reread transcripts, and reworked our interpretations of their interconnectedness. Finally, our writing process clarified and solidified our interpretation and resulted in the discourses presented below.

To enhance the trustworthiness of our analysis, we used critical friend techniques^14^ and a decision trail,^15^ whereby each methodological choice and its rationale was discussed and documented, and any changes or new decisions were included in written correspondence between us to provide an easily traceable record of our process.

## Study Findings

**Figure 1:**
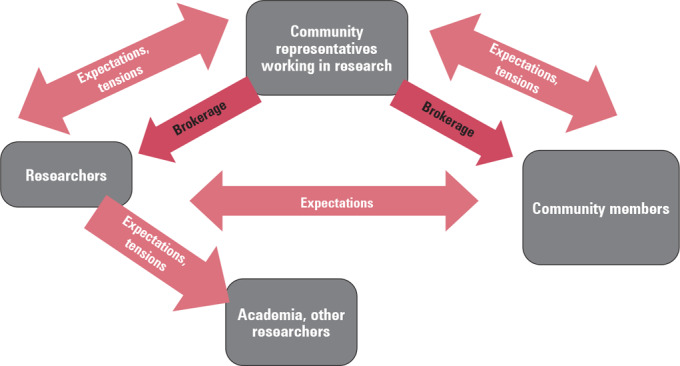
Relationships Between Participant Groups and Discourses

We identified three main discourses that capture the dynamics and interconnectedness of the three participant groups’ perspectives on community engagement. These were expectations, tensions, and brokerage. Each discourse comprised multiple and varied themes and patterns that were partly specific to participant groups and partly shared across the groups. A visual representation of these three discourses (figure 1) illustrates how expectations (both constructive and challenging) featured in the dynamics between all three participant groups, while discourses of tension were prominent but not expressed between researchers and community members. Discourses of brokerage were predominantly constructed by the community representatives as they attempted to bridge the two worlds of academia and community realities. Researchers also expressed expectations and tensions held toward other researchers and academic institutions or practices, and we will discuss this to the extent that it relates to community engagement.

### Expectations

Expectations characterize the dynamics between the different groups, with specific nuances depending on which two groups were considered in relation to each other. Discourses of expectations expressed by researchers and community representatives roughly concerned what could be gained from the other group, whether for themself or for the purposes and interests of research or the wider community.

The community representatives tended to express what they expected from research from the perspective of the community, such as, for instance, improvements they wanted to see in research practices. They also had expectations of communities getting actively involved in research and, for example, helping to reach recruitment targets.

Researchers and community representatives tended to display expectation in relation to their own work or that of other researchers, also revealing the challenges they were grappling with in pursuing ethical and community‐benefitting work.

• **Expectations of research being beneficial**. Community members tended to generally consider research as beneficial or expected it to be valuable in some way. As this quote from a community member illustrates, discourses of expectations concern both research or researchers being impactful and researchers’ presence in communities or their role in relaying information to the community based on their research: “You know, if researchers would be more visible in the community engaging, you know, it will be very effective. And we'll be able to, to understand more of ourselves as human beings …. We need more communication from health to teach us on our health, on how to live” (Interview 9).

These expectations also convey an underlying belief that researchers who have knowledge about health also hold knowledge on “how to live,” giving them enormous power and responsibility. These expectations do not, naturally, predict how evidence or information might be received or what impact they would have, but they do highlight the status that researchers and academics often have from the perspective of community members working to improve things in and for their own communities.

Related to expectations around knowledge and information are expectations around institutional connections that researchers are assumed to have, leading community members to see value in engaging with researchers more generally. Assumptions and expectations about easy or “obvious” access that researchers are believed to have to other researchers, health services, and projects or faculties beyond their own, were both explicitly expressed and implicitly held based on suggestions made regarding future research and interventions. Universities often appear as coherent or unified institutions in the community members’ discourses, where health researchers might easily collaborate with other departments such as engineering, or across health professions or disciplines. As one respondent noted, “Maybe if you were to collaborate like you, you guys are researchers. Obviously, you've got like your doctors, your urologist, your[,] you know, all different types of doctors. … So since you guys do research, maybe if you were to partner with those other institutions with the doctors for them to at least explain it to us in a more simpler way” (Interview 9).

These sentiments highlight the fragmented nature of academic research, and show how the siloes researchers tend to work in are not apparent or logical from the point of view of communities’ needs or interests.

An expectation expressed by a community member representing local government also points to perceptions of research that the mere understanding of a societal or health‐related phenomenon is not enough, that action, such as intervening on drug‐related community problems, is needed more urgently. Interviewer: “So when you recommend that research is done on that, what expectations do you then have of what …. What would that research do? Ideally? Like what would be, in what way could the research be useful and helpful to the community with that issue?” Participant: “Going to ask them to quit rather than waiting around to see what causes these people to take drugs” (Interview 10).

Here, characterizing research as “waiting around” when it seeks to understand a phenomenon is illustrative of how the purposes, processes, and impacts of health research are difficult for community members to observe or can lack credibility from their perspective.

• **Managing expectations**. Community representatives were aware of expectations from both academia/research and communities, and their discourses of expectations centered on navigating or harmonizing those expectations from academia/research and from communities. As this quote from a community representative shows, community members and research participants were also anticipating what researchers expect from them, expressed in this interview as “ulterior motives”: “So obviously, like, especially with the research participants, there's always like [a feeling], ‘what's your ulterior motive’? Right? … you need someone to actually break it down, right? I, yes I'm employed by the research project. However, let me break it down to you, right” (Interview 11).

Similarly, community representatives were used to anticipating the expectations of community organizations (as an example of community stakeholders) toward research. According to one participant: “The challenge that comes to mind when you talk about community organizations, is they always expect something [from research institutions or researchers] out of their participation” (Interview 8).

A common theme within discourses of expectation was the need to manage and meet expectations of research. Apart from understandably expecting benefits or compensation, expectations among research participants were commonly related to receiving feedback about research. Community representatives expressed frustration at not always being able to meet such expectations due to the project‐based nature of health research. Misunderstandings often arose among research participants, even when information given about the research had seemingly been clear regarding the extent of feedback participants could expect to receive. Strategies to manage and meet expectations as best as possible were therefore necessary. Said one participant, “And even if it won't be like individual results, but try to engage with your participants, let them know, … what's the status of the data‐collection. … And if they ask you a question, you must be able to answer. If you can't answer now, just assure them you'll get back to them and make it their point that by the end of the day, they leave the unit with all the questions answered” (Interview 2).

The importance of keeping promises, and of not making promises that cannot be kept, was similarly stressed across interviews with community representatives. As one community representative explained, “So I think the dynamics to it is just around them knowing what we're doing, and at least getting to trust how we're doing things and as just keeping our way, you know, don't make promises that you can't keep. Because at the end of the day, it ruins the relationship” (Interview 8). Another community representative expressed a similar view about making promises: “Believe you me, you must be careful when you talk to these kinds of [stakeholders]. You must be smart because they will hold it against you at the meeting if you didn't do what you promised. So whatever you say, you must act on it” (Interview 2).

While these aspects of accountability can be seen as fundamental and obvious to any form of trust‐building, it was clear from the way participants stressed the importance of keeping promises that this was, in fact, not always obvious or regularly practiced in research, linking many discourses of expectations to the tensions that will be described in more detail.

• **Researchers’ unmet expectations**. While many of the expectations different groups expressed or were seen to hold were unmet, such as the more action‐oriented potential that community members viewed research to hold, there was not much resentment or disappointment expressed in the interviews with community members or community representatives. Research was one potential avenue for achieving change, and while disappointment may lead to disengagement, it was not something characterizing the discourses of the two community‐side participant groups. However, researchers who had experience with community engagement or participatory research practices expressed having a hard time coming to terms with the limitations of academic institutions and logics that hindered preferred community engagement practices. These researchers expected or hoped other researchers would share their view of what “true” participatory research or community engagement meant but acknowledged that this was not necessarily the case. One researcher noted that “I don't think that you can claim to be doing community‐based research …. Unless you're a true participatory action [researcher] and you're doing community‐based research where the community really needs the research … and the community has a voice. And that's a very, very different type of research. Very different” (Interview 6).

Expectations toward institutions were often colored by disappointment. One participant explained how engaged research may lead to enthusiasm from colleagues but that institutions do not typically make use of the momentum generated by successful community engagement or participatory research, and so the work continues without community insights resulting in concrete changes: “When we have a completed project … we have an opportunity to feed back about our work within the organization. Everybody is all of a sudden very excited, enthusiastic, saying, you know, ‘this is what the entire organization should be doing.’ … [I]t's like, you know, we were the star of the show. And then we go back after those meetings and then you start to grind away again and forgotten. So it's, it's challenging” (Interview 5).

These unmet expectations are closely linked to the discourses of tensions discussed in more detail below.

### Tensions

Discourses of tension were prominent across different participant groups and captured tensions between groups as well as tensions within academic research, research teams, or community initiatives. We will focus less on the expressions of tension within communities, apart from where they directly link to engagement with research.

• **Researchers holding tension toward other researchers and their practices**. The researchers we interviewed saw themselves as being somewhere in between research and the communities their research was meant to benefit, and their discourses often involved a demarcation between themselves and other researchers who were not as community‐minded in their work. A typical criticism included unawareness of community realities, which is how the researchers positioned themselves as being more in tune, or as having some degree of insider knowledge thanks to their experiences. Critiques were aimed at both South African colleagues and international collaborators, but particularly the latter. For one of the participants, the lack of awareness about community realities was cited as one of the reasons to leave a research position: “They'll say this research will influence policy. Which policy, do you know which policy? Have you ever read the policy? Do you know how the policy has been implemented on the ground? Have you ever gone and spent a week in a hospital or a week in the clinic? Have you spent a week with a community health worker? Do you know what they do? I mean, it's unbelievable to me. Unbelievable. Sorry, I'm going on a rant, but you can see why I left” (Interview 6).

Another researcher described the institutional (including funders’) demands on engagement and research as being what ultimately guides action, and how difficult such a dynamic can be when pursuing partnerships and community engagement: “But I think a big one … is really that we do joint community engagement and yet our KPIs [key performance indicators] are slightly different on just the community engagement outputs. [Name of institution] has different KPIs for community engagement versus the other partners. But we're trying to pursue joint community engagement. And so being able to agree on what is priority and what is not. What activities that we take, even those things become something. And yet we are all under pressure to, you know, to show that we use the funds the right way” (Interview 1).

Here, it is interesting to note the participant's use of the acronym KPIs. While some researcher participants focused on the meaning and nuances of participatory research or community engagement practices, this participant working with a range of nonacademic stakeholders used corporate expressions such as “KPIs” and “community engagement outputs” to describe challenges such as prioritization. This language comes across as more pragmatic than idealistic when contrasted with the earlier discussed unmet expectations and tensions other researchers expressed regarding to community engagement practices within academia.

• **Sitting on a fence between the community and research**. The internal tensions of academic research clearly restricted researchers’ ability to engage in the kind of community‐centered research they wished to conduct, and often left the researchers grappling with ethical and professional tensions. As one researcher noted, “I always felt all the time I was sitting on a fence, you know, and if you jump off the fence and then you're on the side of the community too much. … Then if you jump on the side of the researchers too much, then the community's not getting what it should” (Interview 6). The researcher went on to describe tensions between research activities and prioritizations, such as meeting certain daily targets of recruitment and data collection versus dedicating time to giving participants sufficient information or feedback (in the case of long‐term participation) regarding research: “But, of course, that costs money because then it's more time. So instead of seeing five, five people in a day in the field, we see three because they've got to really explain what the results, what the research is advancing” (Interview 6).

This scenario reveals how the interests of research are often in contention with those of participants or communities, and such challenges become an ethical burden on researchers who feel they are “sitting on a fence.” As one participant explained, “You have to get funding. That's OK. … But it's hard. But you know, that you really are working with the community is not something that I think a unit such as [institution A] or even [institution B] is particularly set up to do. I think that we have to be very honest about researchers, that they also have their careers to look after” (Interview 6).

According to researchers’ discourses, the wider institutional and systemic context of academia could overshadow the motivation and commitment of individual researchers to work in ways they felt important and impactful. This led to frustration, especially among researchers with less power or autonomy within their own organizations. In contrast, some more senior researchers expressed more hopeful sentiments about community‐centered work while acknowledging the challenges of balancing research and community objectives. “The work typically is delivered as per protocol, and there isn't a lot you can do within the study that's often that creative. But I think that the creative space maybe comes after that. Obviously, that takes effort because the finances that pay your time to continue to think about that have dried up as well. So you're forced onto the next opportunity …. So it's definitely not easy, … but I've always found that action is better than inaction and doing something [for the community] and causing trouble [within the institution] and then having to fix it. … You're causing trouble. But you also find you're actually moving somewhere versus worrying about how

**We call for caution and critical consideration of how much we as researchers ask of research participants and communities in our efforts to improve our own practices.**

to do something because you're going to likely cause trouble” (Interview 5). This interviewee suggested that by “causing trouble,” they'd managed to make progress. “OK, well, … something's going to go wrong. But let's do something. Let's act and see how we can respond once we see what's come of that” (Interview 5).

The way this more senior researcher speaks about opportunities to “cause trouble” (as a way to challenge the status quo to make positive change) and about how they make progress once formalities have been fulfilled is likely at least partly reflective of the longer experience and professional security they have been able to achieve within an academic institution. Seniority may thus afford a higher degree of autonomy and “creativity” in terms of incorporating community‐centered action into research.

• **Rigidity of research practices**. Community representatives working in research also described tensions between themselves or their work and the typical practices of researchers or academic research. For example, the project‐based nature of research and the lack of continuity in research‐community relationships were a source of tension and frustration, and community representatives working in research had tried to think of ways to improve the situation. According to one participant, “I feel … we can improve on and make it a point that we stay in contact with these people” (Interview 2).

A paper trail and other elements of institutional memory and coordination were clearly lacking in the project‐based research practices described by participants, and this often led to repeated or duplicated efforts.

More generally, participants representing both communities and research tended to point out the shortcomings of processes laid out in advance when working in complex and dynamic environments. While acknowledging the importance of institutional requirements such as standard operating procedures (SOPs) and ethical standards like Good Clinical Practice (GCP), this participant emphasized the need for flexibility within boundaries: “Have a picture of how everything will be done, then revise it as you start with your pilot. Because whatever you always have on paper, it's not exactly how it's going to work out there. So we will, researchers, we tend to type everything, following ethics, following whatever knowledge we have in our heads, then, when we get to the field, you'll find that not, that procedure that you've said you take might not really yield the best result at all. So at least get your team to know the GCP rules so that even if they have to adapt, they may not deviate from the SOP and protocol” (Interview 8).

Navigating a delicate balance between key institutional frameworks and the realities of fieldwork will be further examined under discourses of brokerage.

### Brokerage

Discourses of brokerage were expressed by community representatives as they grappled with the varying or even conflicting expectations of different actors involved in research and community engagement. These expectations often manifested in the way they would try to reconcile community needs with rigid research practices, but also in how they made efforts to ensure community members and potential research participants really understood what the purpose and process of a research project was.

• **Getting communities onboard**. Similar to the use of corporate terms like KPIs by researchers under pressure from funders, a community representative also talked about navigating the research‐community relationship and particularly recruitment through, initially, a sales approach centered on the needs of research, but how they later realized that would not be compatible with the needs and interests of participants. As one participant pointed out, “It's not a sales environment, but the approach is sort of a sales pitch, right? ‘This is the product that I have, do you want to take part and interact?’” (Interview 11).

While it was unclear whether the sales approach was encouraged by project superiors, the participant went on to elaborate on how they had reflected on the appropriate approach to engaging with potential research participants, and formed practices that were in line with their personal ethics in representing both research and community perspectives: “We should not see participants as subjects, but as actual people … because if we humanize the research project, then we can definitely actually like assess like ‘is what I'm doing to the participant or from the participant, is that in line with my ethics and whatnot? Yes, yes, it does.’ Okay, then carry it out, right? … And if you're working with that kind of context, means that it's a chance to change, the research environment needs to change, so that we enable participants to be willing at any time to withdraw or refuse or whatnot” (Interview 11).

In addition to grappling with the ethical challenges of research and recruitment, there were also many other examples of how the work done by community representatives within research projects involved constantly adapting and developing one's approach, and this kind of problem‐solving was a typical example of brokerage they engaged in because the solutions were not provided by the projects’ senior leadership. “When COVID happened,” said one participant, “it became very difficult to engage with communities. So I had to come up with another strategy which is engaging with community members by engaging, finding someone within the community who they know in that community to assist you … So, with the help of the community, I start seeing the wheels [of recruitment] turning. … So those are the kind of big challenges that you encounter convincing people in the community” (Interview 2).

Indeed, this participant's description of engaging community members, enlisting their help for recruitment, and ultimately “convincing people in the community,” demonstrates the kind of brokerage strategies that community representatives working in research engage in, whereby they are not necessarily the right people themselves to reach enough people, but they make use of their connections to fulfill research targets. In this case, the participant also talked about how the compensation that they were able to provide to community members assisting in recruitment was beneficial, and thus how their brokerage also involved leveraging research resources for the benefit of others in the community: “So it also gives back to the community and in some instances, because like, they're able to put the bread [on the table] at the end of the day. So, yeah, it's very helpful” (Interview 2).

However, skillful brokerage is also required for navigating politically sensitive relationships and for managing the expectations around one's ability to “leverage” research resources. Specific expectations may arise from engaging stakeholders such as local politicians, who often act as gatekeepers in terms of community entry or other aspects of relationship building with communities. One participant pointed out that “You meet with a ward councilor, and with ward councilors generally, there's also like, the word that I used, ulterior motives, right. So there's gatekeepers. And there's the political in the sense that because in urban and rural areas, right, it's highly political. So whatever you need to do, you need to get permission and support. … It's kind of like ‘Scratch, scratch, scratch your back, I scratch your back.’ Right. And from our aspect, we can't, can't scratch it, we don't have the leverage to scratch your back” (Interview 11).

Pursuing reciprocity in research‐community relationships becomes particularly challenging when it may be understood to involve political favors rather than more simply “giving back to communities.” “Ulterior motives” can thus come up in a more compromising way. The language around “scratching someone's back” alludes to exchanging favors in ways that are outside of what can ethically or practically be done in the context of academic research. This highlights the need for brokers to adopt different strategies of engagement than would perhaps be locally relevant in other types of activities, or from what they know is expected of them.

• **Advocating for participants and communities**. Through discourses of brokerage, community representatives expressed ways to ensure that participants and communities were heard and their interests served. Despite not having worked in research very long, one of the participants reflected on their role in pushing for certain practices they knew were of importance to the community and participants: “I've been pushing it with this one, that, ‘can we please’, because that was the most concern we got from participants. ‘People never come back and give us results’, right? And I'm like, ‘can we please definitely ensure’, as much as we've been explaining that they won't get individual results, but something back, just to tell them that, ‘hey, you know what, you took part in this project. As you know, we're not giving like individual feedback, but this is what the study found out” (Interview 11). The community representative went on to explain that it wasn't a lot of information, “but it's something for participants, right, to give them something that ‘actually, I contributed towards something’. So it's something like that” (Interview 11).

This kind of brokerage illustrates how respecting community priorities may require personal efforts beyond one's job description, and how relatively junior research team members brave the challenges of research hierarchies. The participants’ accounts revealed that the prevailing practices are rarely set up to ensure that community preferences or interests are served. As a result, community representatives working in research often made extraordinary efforts to give back to participants or communities in ways they felt were appropriate and warranted. Community engagement was typically seen as the natural avenue for bringing the benefits of research closer to communities and potentially even inspiring community‐led action through research insights and encounters. “If you have community engagement where you can just bring insight and say, ‘OK, can we try this?’ It doesn't have to work, but at least we've tried it. It makes people kind of always want to think of how to better their communities, you know? … You have to have more community building, community engagement interventions where people can somehow take ownership. … So it's no small thing where you like ‘what, what can I do to just bring about the change?’ You know? But I think there is no solid structure, especially in [the research institution], to just put like emphasis on community building because it's looked upon as a small initiative. But I think if you get it right today, who knows what you can do?” (Interview 7).

These reflections about how research and community engagement could play a role in “community building” and “bringing about change” also illustrate the personal burden of navigating academic research while being crucially aware of community needs, which may not be the case for colleagues who are less embedded in the communities involved in research.

While there were some examples among researcher participants of developing ways to achieve outcomes that serve the interests of communities by changing or challenging academic practices (“causing trouble” or changing “the research environment”), the discourses of brokerage expressed by community representatives tended to be more about working within academic systems and projects through making the most of what is possible. Discourses of brokerage thus capture the balance that community representatives working in research seek between fulfilling their job requirements and bringing the benefits of research to their communities. As this final quote summarizes, the desired impact will take time and perseverance: “I think research is an amazing thing. … It's a space that we need more and more of. It is an amazing thing. It's, I don't think we should, because we are not winning as we thought we would, just close the whole shop. No, I just think it will take time” (Interview 7).

## Discussion

In our multiperspective discourse analysis, we identified three connected discourses of expectations, tensions, and brokerage. These discourses are used somewhat differently by different participants who have different relationships with research and community engagement. While participants often constructed discourses around difficult aspects of research and community engagement, highlighting both institutional and structural challenges, their ways of responding to expectations and tensions also revealed hopefulness (e.g., opportunities for “causing trouble”) and tactical brokerage (e.g., bringing research benefits to communities to satisfy both project demands and community expectations).

A recent systematic review of ethical challenges affecting health research staff in low‐ and middle‐income countries highlights the impacts of disadvantaged research participants’ requests for help, and related feelings of guilt among the issues research staff face through their work.^16^ We found such challenges to be present in the discourses constructed by both researchers and the research staff representing the communities they work in as well. While we also have personal experience of navigating the expectations and tensions related to this from the perspective of “outsider” researchers, we want to stress the added pressures such factors place on colleagues who face expectations from multiple directions,^17^ and who are often left in the role of broker, mitigating conflicts and tensions that others can perhaps more easily switch off because they don't have personal ties to the communities in question.

There are calls for more transformative, power shifting approaches to community engagement and community‐based research in general,^18^ but without significant institutional changes, brokerage becomes the main solution for managing the relationships between research teams and communities, characterized by expectations and tensions. As our discourse analysis shows, this puts the onus on the brokers—the actors who represent both parties or who try to harmonize and navigate the dynamics between researchers and participants due to their own ethical commitment. Similarly, researchers committed to doing “true” community engagement or participatory research are torn between the different expectations and tensions arising from interests that are rarely fully aligned.

Our analysis also pointed to positive experiences and sources of hope, particularly relating to researchers or research teams finding ways to incorporate community‐benefitting activities, service provision, or other ways of giving back to communities. These are not perfect solutions, and they feed back into the discourses of expectations, as they strengthen the image of research organizations having unlimited resources or power. However, through committed efforts, and particularly through long‐term research initiatives that achieve some continuity or cause progressive “trouble” in the settings where they work, researchers felt they could contribute more to communities than the research alone. A previous critical discourse analysis of participatory research located practices along a continuum from instrumental to transformative.^19^ This seems to be one of the key tensions researchers we interviewed point to when expressing their disapproval of other researchers’ practices, that the research practice was more instrumental than transformative, which went against the expectations the researchers held about their own and others’ work with communities.

The phenomenon of brokerage has been extensively theorized and studied in development studies and other fields,^20^ and its implications for health research has also been examined in the context of, for example, broker‐facilitated interviewing or recruitment procedures, with ethical and methodological implications.^21^ In our analysis, we found the discourse of brokerage to cover a number of relationships and practices, but it primarily covers the actions of community representatives working in research trying to harmonize the interests and practices of research institutions and communities. The limitations of brokerage were also evident in the discourses of community representatives involved in research. As the example of “back‐scratching” illustrates, skillful brokerage involves recognizing the limits of action when representing research institutions, even if it may go against the logic of getting certain actors on board. In view of developing a future ethics of brokerage, there is scope for more research to examine and theorize around different typologies and limitations of brokerage in community engagement.

As with the review of ethical dilemmas,^22^ brokerage has been particularly described as a form of negotiation in the context of North‐South development practice, but in our research in South Africa, we see similarities in power dynamics due to the country's history of racial oppression and current inequalities that still largely follow the patterns of apartheid. Senior researchers without lived experience from, or personal ties to, participating communities are often in similar positions vis‐à‐vis the communities as foreign researchers from the global North, and brokerage becomes the responsibility of research staff representing the communities in question.^23^ In practice, (improvised) brokerage fulfills the purpose of community engagement in many situations where research institutions lack the foresight or capacity to pursue community engagement in a more organized and coherent way. This is because improvised brokerage fills the gap of intentional relationship building. However, while it may work well in practice, this brokerage places undue pressure on the research staff who are able to successfully broker these relationships, and seldom involves appropriate recognition or compensation for work done beyond what research protocols capture. Some brokerage would likely occur even under more favorable institutional conditions, and it need not automatically be problematized. Reliance on individuals to make up for a lack of institutional support is what we find ethically and practically concerning.

The need for brokerage to facilitate successful community engagement is in line with a recent systematic review of community engagement in research in Sub‐Saharan Africa,^24^ which identified factors such as specific cultural, historical, and religious practices, as well as communication challenges, as key barriers to engagement. Overcoming such barriers understandably requires or benefits from skillful brokers who can navigate the rules and logics of both academic research projects and local communities. While providing a crucial solution to community engagement challenges, community representatives thus easily end up, through their skillful brokerage, standing in where institutions fall short.

Compared to community representatives and researchers, the community members participating in our study expressed more optimistic expectations and less cynical reflections around the impact that research can have. However, we recognize that we had an easier time analyzing the discourses of research‐side participants than community members due to our own positionalities and ability to relate to the ethical and professional conundrums they face. This was a shortcoming in our study and limited the extent to which we felt comfortable interpreting the more latent content of community member interviews. Our analytic output centered on the perspectives of researchers and community representatives.

We have reflected on how it is important to ensure that community perspectives are effectively incorporated into research processes, but at the same time, we cannot as researchers expect community members to fix the problems of academic institutions. As such, we find it challenging to balance the degree to which community views are solicited, since community members not involved or experienced in research may, understandably, have little interest or practical capacity (e.g., available time) to inform research practices. We have found practices such as scaffolding through specific questions and prompts to be helpful in gaining feedback in such situations, but we call for caution and critical consideration of how much we as researchers ask of research participants and communities in our efforts to improve our own practices. While these learnings are highly relevant in South Africa, we believe they have relevance beyond our research context. Community engagement—and the importance of effectively capturing community perspectives—is critical in all global settings, but especially where structural factors and unfair knowledge practices have historically contributed, or continue to contribute, to the devaluing of local and community perspectives.^25^ Furthermore, the related challenges experienced in academic research and institutions are not unique to South Africa, or even the global South,^26^ and community members should not have to bear the burden of these challenges in any settings.

## Acknowledgments

The work of SK was supported through grants 201901846 and 202105895 from Kone Foundation (Koneen Säätiö). We are grateful to our research participants for contributing their time and insights to this study, and to our colleagues at Developmental Pathways for Health Research Unit for inspiring discussions around these topics.

## References

[1] Bain, L. E. , et al., “Community Engagement in Research in Sub-Saharan Africa: Current Practices, Barriers, Facilitators, Ethical Considerations and the Role of Gender—a Systematic Review,” Pan African Medical Journal 43 (2022): 152; Reynolds, L., and S. Sariola, “The Ethics and Politics of Community Engagement in Global Health Research,” *Critical Public Health* 28, no. 3 (2018): 257-68. 36785694 10.11604/pamj.2022.43.152.36861 PMC9922083

[2] Klingberg, S. , et al., “Engaging Communities in Non-Communicable Disease Research and Interventions in Low- and Middle-Income Countries: A Realist Review Protocol,” BMJ Open 11, no. 7 (2021): doi:10.1136/BMJOPEN-2021-050632. PMC8296813 34290072

[3] Yuan, M. , “Community Engagement in Public Health: A Bibliometric Mapping of Global Research,” Archives of Public Health 79 (2021): doi:10.1186/s13690-021-00525-3. PMC7801880 33436063

[4] Klingberg, S. , et al., “Enhanced or Hindered Research Benefits? A Realist Review of Community Engagement and Participatory Research Practices for Non-Communicable Disease Prevention in Low- and Middle-Income Countries,” BMJ Global Health 9, no. 2 (2024): doi:10.1136/BMJGH-2023-013712. PMC10862340 38341191

[5] Abimbola, S. , “Beyond Positive a Priori Bias: Reframing Community Engagement in LMICs,” Health Promotion International 35, no. 3 (2020): 598–609; Burgess, R. A., “Working in the Wake: Transformative Global Health in an Imperfect World,” *BMJ Global Health* 7, no. 9 (2022): doi:10.1136/BMJGH-2022-010520. 30982066

[6] Kowal, S. P. , T. Bubela , and C. Jardine , “Experiences in Broker-Facilitated Participatory Cross-Cultural Research,” International Journal of Qualitative Methods 16, no. 1 (2017): doi:10.1177/1609406917706883.

[7] Kalinga, C. , “Caught Between a Rock and a Hard Place: Navigating Global Research Partnerships in the Global South as an Indigenous Researcher,” Journal of African Cultural Studies 31, no. 3 (2019): 270–72.

[8] Mthembu, Z. , M. Chimbari , and M. Macherera , “Facilitating Community Engagement: Researchers’ Lived Experiences in Rural Communities in the KwaZulu-Natal Ecohealth Program, South Africa,” Cogent Social Sciences 9, no. 1 (2023): doi:10.1080/23311886.2023.2225833.

[9] Draper, C. E. , et al., “The Role of Partnerships to Shift Power Asymmetries in Research with Vulnerable Communities: Reflections from an Early Childhood Development Project in South Africa,” Journal of Cognition and Development 25, no. 2 (2023): 222–41.

[10] Cumyn, A. , et al., “Role of Researchers in the Ethical Conduct of Research: A Discourse Analysis from Different Stakeholder Perspectives,” Ethics & Behavior 29, no. 8 (2019): 621–36; Ziskin, M. B., “Critical Discourse Analysis and Critical Qualitative Inquiry: Data Analysis Strategies for Enhanced Understanding of Inference and Meaning,” *International Journal of Qualitative Studies in Education* 32, no. 6 (2019): 606-31; Collins, P. H., et al., “Intersectionality as Critical Social Theory,” *Contemporary Political Theory* 20, no. 3 (2021): 690.

[11] Braun, V. , and V. Clarke , “To Saturate or Not to Saturate? Questioning Data Saturation as a Useful Concept for Thematic Analysis and Sample-Size Rationales,” Qualitative Research in Sport, Exercise and Health 13, no. 2 (2021): 201–16; Morse, J. M., “The Significance of Saturation,” *Qualitative Health Research* 5, no. 2 (1995): 147-49.

[12] Cumyn et al., “Role of Researchers in the Ethical Conduct of Research.”

[13] Klingberg, S. , et al., “‘Must You Make an App?’ A Qualitative Exploration of Socio-Technical Challenges and Opportunities for Designing Digital Maternal and Child Health Solutions in Soweto, South Africa,” PLOS Global Public Health 2, no. 12 (2022): doi:10.1371/JOURNAL.PGPH.0001280; Klingberg, S., R. E. Stalmeijer, and L. Varpio, “Using Framework Analysis Methods for Qualitative Research: AMEE Guide No. 164,” *Medical Teacher* 46, no. 5 (2023): 603-10. PMC10021787 36962834

[14] Smith, B. , and K. R. McGannon , “Developing Rigor in Qualitative Research: Problems and Opportunities within Sport and Exercise Psychology,” International Review of Sport and Exercise Psychology 11, no. 1 (2018): 101–21.

[15] Cheek, J. , “At the Margins? Discourse Analysis and Qualitative Research,” Qualitative Health Research 14, no. 8 (2004): 1140–50. 15359048 10.1177/1049732304266820

[16] Steinert, J. I. , et al., “A Systematic Review on Ethical Challenges of ‘Field’ Research in Low-Income and Middle-Income Countries: Respect, Justice and Beneficence for Research Staff?,” BMJ Global Health 6, no. 7 (2021): doi:10.1136/BMJGH-2021-005380. PMC8292801 34285041

[17] Kalinga , “Caught Between a Rock and a Hard Place.”

[18] Klingberg et al., “Enhanced or Hindered Research Benefits?”; Burgess, “Working in the Wake”; Klingberg et al., “‘Must You Make an App?’”

[19] Jacques-Aviñó, C. , et al., “Are We Leaving Someone Behind? A Critical Discourse Analysis on the Understanding of Public Participation among People with Experiences of Participatory Research,” PLoS ONE 17, no. 9 (2022): doi:10.1371/JOURNAL.PONE.0273727. PMC9439240 36054140

[20] Lewis, D. , and D. Mosse , Development Brokers and Translators: The Ethnography of Aid and Agencies (West Hartford, CT: Kumarian Press, 2006).

[21] Kowal, Bubela , and Jardine , “Experiences in Broker-Facilitated Participatory Cross-Cultural Research.”

[22] Steinert et al., “A Systematic Review on Ethical Challenges of ‘Field’ Research in Low-Income and Middle-Income Countries.” 10.1136/bmjgh-2021-005380 PMC8292801 34285041

[23] Kalinga , “Caught Between a Rock and a Hard Place.”

[24] Bain et al., “Community Engagement in Research in Sub-Saharan Africa.”

[25] Abimbola, S. , et al., “Unfair Knowledge Practices in Global Health: A Realist Synthesis,” Health Policy and Planning 306 (2024): doi:10.1093/HEAPOL/CZAE030. PMC11145905 38642401

[26] Jagosh, J. , et al., “A Realist Evaluation of Community-Based Participatory Research: Partnership Synergy, Trust Building and Related Ripple Effects,” BMC Public Health 15, no. 1 (2015): 725. 26223523 10.1186/s12889-015-1949-1 PMC4520009

